# Cellular and molecular remodelling of a host cell for vertical transmission of bacterial symbionts

**DOI:** 10.1098/rspb.2016.0580

**Published:** 2016-06-29

**Authors:** Jun-Bo Luan, Hong-Wei Shan, Philipp Isermann, Jia-Hsin Huang, Jan Lammerding, Shu-Sheng Liu, Angela E. Douglas

**Affiliations:** 1 Department of Entomology, Cornell University, Ithaca, NY 14853, USA; 2 Meinig School of Biomedical Engineering and Weill Institute for Cell and Molecular Biology, Cornell University, Ithaca, NY 14853, USA; 3 Department of Molecular Biology and Genetics, Cornell University, Ithaca, NY 14853, USA; 4 Ministry of Agriculture Key Laboratory of Agricultural Entomology, Institute of Insect Sciences, Zhejiang University, Hangzhou 310058, People's Republic of China

**Keywords:** bacteriocyte, *Bemisia tabaci*, cell mobility, *Portiera*, vertical transmission

## Abstract

Various insects require intracellular bacteria that are restricted to specialized cells (bacteriocytes) and are transmitted vertically via the female ovary, but the transmission mechanisms are obscure. We hypothesized that, in the whitefly *Bemisia tabaci*, where intact bacteriocytes (and not isolated bacteria) are transferred to oocytes, the transmission mechanism would be evident as cellular and molecular differences between the nymph (pre-adult) and adult bacteriocytes. We demonstrate dramatic remodelling of bacteriocytes at the developmental transition from nymph to adulthood. This transition involves the loss of cell–cell adhesion, high division rates to constant cell size and onset of cell mobility, enabling the bacteriocytes to crawl to the ovaries. These changes are accompanied by cytoskeleton reorganization and changes in gene expression: genes functioning in cell–cell adhesion display reduced expression and genes involved in cell division, cell motility and endocytosis/exocytosis have elevated expression in adult bacteriocytes, relative to nymph bacteriocytes. This study demonstrates, for the first time, how developmentally orchestrated remodelling of gene expression and correlated changes in cell behaviour underpin the capacity of bacteriocytes to mediate the vertical transmission and persistence of the symbiotic bacteria on which the insect host depends.

## 1. Introduction

All animals are colonized by microorganisms that promote the health and fitness of their animal host [1]. Many associations are open, meaning that microorganisms can be gained by the animal from external sources and are shed from the animal back to the environment, but other associations are closed with obligate vertical transmission, generally from mother to offspring [2]. Among the best-studied closed systems are the bacteriocyte symbioses of insects, in which bacterial symbionts are restricted to a single cell type, the bacteriocyte, and transmitted vertically via the female ovary, usually by insertion directly into each oocyte [3]. This process is known as transovarial transmission. These associations have evolved independently multiple times, including in various pests of agricultural and medical importance [3,4]. The patterns of vertical transmission of bacteriocyte symbionts vary widely among different insect groups, especially with respect to the time that the bacterial symbionts spend in the extracellular condition between exit from the maternal bacteriocyte and entry to the oocyte [3,4]. Although transovarial transmission is crucial to the persistence of the symbiosis on which the insect depends, the underlying mechanisms are largely unknown and generally considered to be intractable to analysis.

We reasoned that the remarkable mode of transmission in one group of insects, the whiteflies, offers a unique opportunity to investigate the mechanism of transmission. In whiteflies, intact bacteriocytes are transferred to the ovaries of the adult female without any extracellular phase of the symbionts [3,5,6]. Because the bacteria are not released from the maternal bacteriocyte during transmission, functional traits related to transmission should be evident as differences in the properties of bacteriocytes in the adult female (which are transmitted) and bacteriocytes in the nymph (pre-adult) stage (which are not transmitted).

Our specific hypothesis was that the cellular behaviour and gene expression patterns differ between bacteriocytes in adult, reproductive females and nymphs of whiteflies in ways that are congruent with the pattern of vertical transmission in whiteflies [3,5,6]. We tested this hypothesis with the silverleaf whitefly *Bemisia tabaci* MEAM1, which is a globally important agricultural pest [7]. As in other whiteflies (family Aleyrodidae), the bacteriocyte symbiont is *Candidatus* Portiera aleyrodidarum (henceforth *Portiera*) [8], which provides the insect host with essential amino acids, nutrients in short supply in the whitefly diet of plant phloem sap [9]. Typically, *B. tabaci* bacteriocytes bear a second bacterial symbiont, the identity of which varies among species; the *B. tabaci* MEAM1 studied here bears *Hamiltonella defensa*. In this system, the bacteriocytes (and no other insect cells) are green–yellow, probably due to carotenoids synthesized by the *Portiera* [10], facilitating their identification and enumeration.

We reveal that the whitefly bacteriocyte is a remarkably dynamic cell type that changes from a non-motile, adherent condition in nymphs to motile cells that continue to proliferate through adulthood, generating a continuous supply of cells for vertical transmission. These changes in cell behaviour are underpinned by major differences in the expression of genes for cell adhesion and mobility, providing the molecular basis for the transmission process.

## 2. Material and methods

### (a) Insects

The whitefly *B. tabaci* MEAM1 cultures were maintained in climate-controlled chambers at 27±1°C with 14 L : 10 D regime.

### (b) Bacteriocyte dynamics

The number of bacteriocytes was scored in 10 individuals dissected in phosphate-buffered saline (PBS) at pH 7.4 for 1- to 5-day-old eggs, third instar nymphs, early stage of fourth instar nymphs (prior to detectable eye pigmentation), late stage of fourth instar nymphs (with red eyes, known as pupae) and female adults at 0–35 days after emergence. The diameter of bacteriocytes in each sample was determined using an eye-piece graticule in a Leica stereo microscope and cell volume was calculated as 4/3*πr*^3^. The bacteriocytes from 7-day-old adults used for analysis of shape were videoed at 500× magnification under a Keyence VHX-2000 digital microscope. Time-lapse photography was conducted at one frame per 15 s over 10 min for 10 replicate bacteriocytes, and area (*A*), perimeter (*P*) and centre of mass were determined by image analysis software ImageJ. Cell circularity was calculated as 4*πA*/*P*^2^ and migration distance was determined by centre of mass. Bacteriocyte uptake by ovarioles used ovaries isolated into PBS from adult females 0–7 days after emergence.

### (c) Microscopical analysis

The localization of *Portiera* and *Hamiltonella* in whiteflies was investigated by fluorescence *in situ* hybridization (FISH) with the probes BTP1-Cy3 (5′-Cy3-TGTCAGTGTCAGCCCAGAAG) for *Portiera* and BTH-Cy5 (5′-Cy5-CCAGATTCCCAGACTTTACTCA) for *Hamiltonella* [11]. Stained samples were viewed under a Zeiss LSM780 confocal microscope. To visualize the cytoskeleton, bacteriocytes and ovarioles were fixed and permeabilized. For the first assay with nymph bacteriocytes and adult bacteriocytes, the samples were blocked and then incubated sequentially with mouse anti-β-tubulin monoclonal antibody (Sigma), goat anti-mouse antibody conjugated to Alexa Fluor 488 (Sigma), and, finally, with phalloidin-Alexa Fluor 568 (Thermo Scientific) and Hoechst 33342 (Thermo Scientific). Microtubule and actin intensities were quantified by ImageJ. For the second assay with ovarioles and adult bacteriocytes, only actin was studied by incubation of samples with phalloidin-Alexa Fluor 488 and 568, respectively. To visualize the membranes in ovarioles with internalized bacteriocytes, ovarioles were incubated in Grace's Insect Medium (Sigma) with FM 4-64 (Thermo Scientific) and Hoechst 33342. Images were collected and analysed on a Zeiss LSM700 confocal microscope.

### (d) RNA-seq analysis

Approximately 20 000 bacteriocytes were dissected from each of approximately 4000 fourth-instar nymphs and approximately 3000 female adult whiteflflies at 7 days after emergence, using fine pins and a dissecting microscope. RNA isolation, library preparation and sequencing were conducted in accordance with Luan *et al.* [9]. We used the pipeline of Luan *et al.* [9] for transcriptome assemblies and differential expression gene analyses. Web gene ontology annotation plot was used to investigate the distribution of gene functions for two samples.

### (e) Statistical analysis

Statistical significance was evaluated using one-way ANOVA at a 0.05 level. Fisher's least significant difference tests were followed for the number of bacteriocyte and egg and Kruskal–Wallis test for bacteriocyte volume. All data analyses were conducted using the software Statistica v. 6.1 (StatSoft, Inc., Tulsa, USA).

See electronic supplementary material, text S1 for complete details on material and methods.

## 3. Results

### (a) Bacteriocyte dynamics in the nymph and adult host

**Figure 1.**
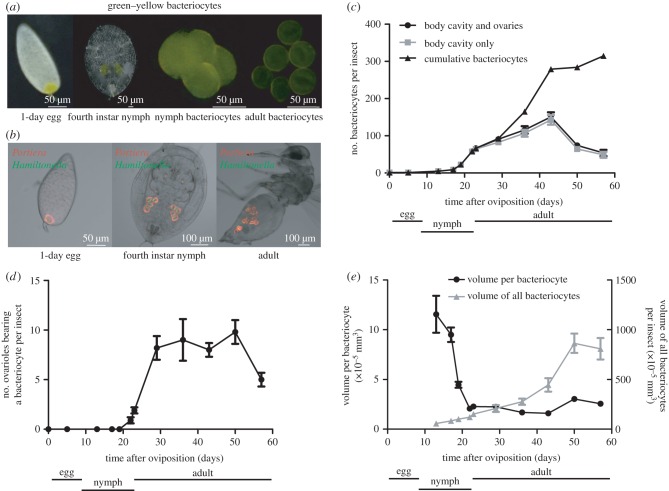
Bacteriocyte dynamics through whitefly lifespan. (*a*) Green–yellow bacteriocytes in the egg and nymph of whiteflies, and dissected bacteriocytes in nymphs and adults. (*b*) The whole-mount FISH of symbiotic bacteria *Portiera* (red) and *Hamiltonella* (green) in different developmental stages of *Bemisia tabaci*. (*c*) Dynamics of bacteriocyte number in the whiteflies through development. *F*_10,99_ = 54.63, *p* < 0.0001 for the whole body and *F*_10,99_ = 47.094, *p* < 0.0001 for body cavity only, respectively. (*d*) Number of ovarioles bearing a bacteriocyte during whitefly development. *F*_10,99_ = 8.176, *p* < 0.001. (*e*) Volume per bacteriocyte and of the total complement of bacteriocytes per insect during whitefly development. *F*_10,99_ = 28.056, *p* < 0.0001 for volume per bacteriocyte. The data in (*c*–*e*) are expressed as mean ± s.e.m. with 10 replicates.

To test our hypothesis that the cellular behaviour of bacteriocytes differs between the adult host (in which bacteriocytes are transmitted to the ovaries) and nymphal host (with no transmission), we quantified the number and size of bacteriocytes through development of the host. This analysis was facilitated by the distinctive green–yellow colour of the carotenoid pigments expressed exclusively in bacteriocytes at all stages of the host life cycle (figure 1*a*). Every egg examined over the first 5 days of the 7-day egg stage contained a single bacteriocyte (figure 1*a*). The number of bacteriocytes subsequently increased through nymphal development to 22.4 ± 1.75 (mean ± s.e.m., 10 replicates) cells in the ‘pupal’ stage (the non-feeding final phase of the fourth nymphal stadium at day 12 after egg hatch). This is equivalent to 4–5 divisions between the day 5 egg and pupa. Bacteriocyte proliferation continued in the adult stage of females to a maximum of 150 ± 12.5 at 21 days after adult emergence. Parallel microscopical analysis revealed that the bacteriocytes in the nymphs comprised two small aggregations, in which part of the surface of every cell was in contact with the external environment, but were loosely packed or spatially isolated as single cells in the adults (figure 1*a,b*).

Bacteriocytes were associated with the ovaries of female insects within 24 h of adult emergence (day 22 in figure 1*c*,*d*). All the ovarioles (the egg tubes in the insect ovary) with a mature oocyte and some ovarioles with immature oocytes bore a single distal bacteriocyte, with up to 7–11 bacteriocytes associated with the ovaries during the period of maximal egg production (7–21 days post-emergence) (figure 1*d*). Averaging across the six time points (1, 7, 14, 21, 28 and 35 days post-emergence) of assay for adult insects, 8.7 ± 0.017% of bacteriocytes were associated with the ovaries. Because each deposited egg bore a single bacteriocyte, the cumulative production of bacteriocytes in an adult female could be estimated from the sum of the number of bacteriocytes in the body and number of eggs deposited over the previous time period (electronic supplementary material, figure S1). The mean of 22.4 bacteriocytes in the pupa had given rise to 279 bacteriocytes by day 21 of adulthood and 315 bacteriocytes at day 35, indicative of 4–5 rounds of cell division in the adult female (figure 1*c*). The mean lifetime allocation of bacteriocytes to transmission was estimated from the mean total reproductive output per insect, at 384 eggs and bacteriocytes (electronic supplementary material, figure S1).

To investigate how the proliferation of bacteriocytes affected the total biomass of bacteriocytes in the association, we conducted a parallel analysis of the size of bacteriocytes. The volume per bacteriocyte varied with host age (Kruskal–Wallis test: *H*_9_ = 74.49, *p* < 0.001), declining more than five-fold from 11.6 ± 1.87 × 10^−5^ mm^3^ (mean ± s.e.m., 10 replicates) in third-instar nymphs to 2.1 ± 0.22 × 10^−5^ mm^3^ in newly emerged adult females, but did not vary significantly through adulthood to day 21 post-emergence (figure 1*e*). The linear regression of mean total volume of bacteriocytes on time was significant (*F*_1,6_ = 58.96, *p* < 0.001, *r*^2^ = 89.2%), yielding net increase of 6.4 × 10^−5^ mm^3^ d^−1^ (figure 1*e*). These results indicate that the volume of the symbiosis increased with host age, both in the growing nymph and in the adult host, which does not increase in size. As considered further in the discussion below, these results suggest that the bacteriocytes in the adult function not only as the vehicle for transmission, but also in the nutrition of the host.

**Figure 2.**
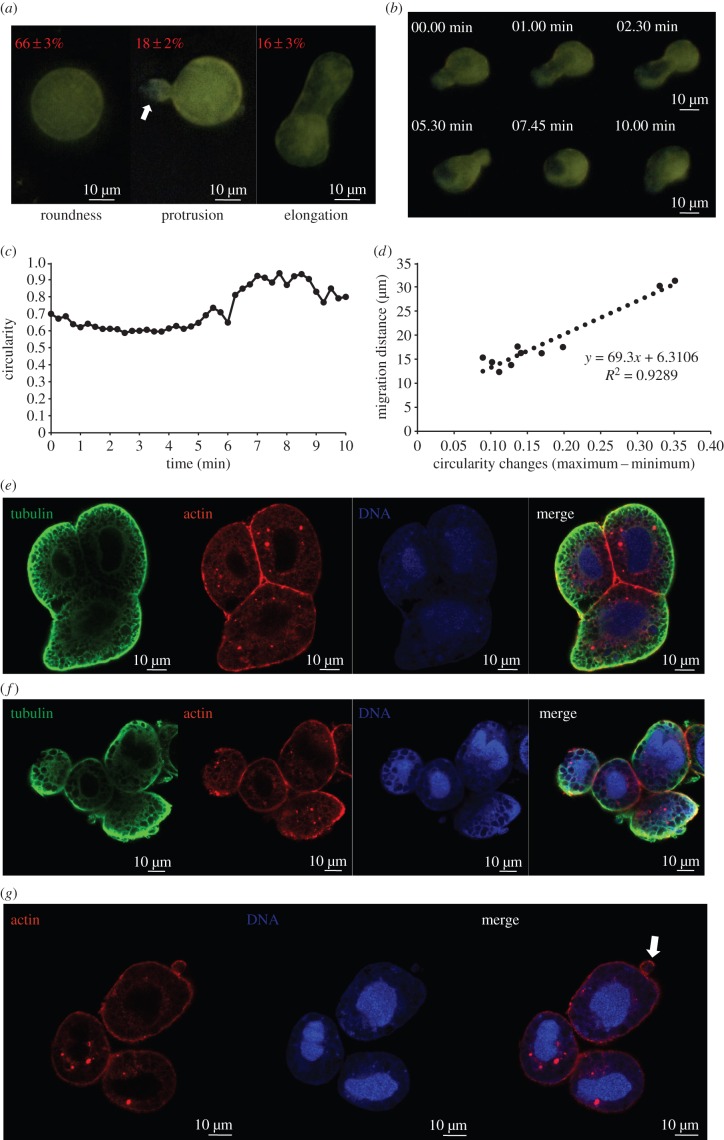
Shape changes and cytoskeleton distribution of bacterioctyes. (*a*) Adult bacteriocytes show round, protrusional (white arrow) and elongated shapes, with percentage of bacteriocytes dissected from 10 replicate 7-day-old adults with each shape (mean ± s.e.m.) shown. (*b*,*c*) Time-lapse sequences of (*b*) cell shape changes and (*c*) circularity dynamics over 10 min. (*d*) Correlation between migration distance and circularity changes of 10 replicate bacteriocytes over 10 min. (*e,f*) Actin (phalloidin-Alexa Fluor 568, red) and tubulin (green) in (*e*) nymph bacteriocytes and (*f*) adult bacteriocytes. (*g*) Actin (phalloidin-Alexa Fluor 568, red) in adult bacteriocytes with protrusion (arrow). DNA was stained by Hoechst (blue).

As we were quantifying the number and size of bacteriocytes, we noted that the bacteriocytes in nymphs and pupae were invariably regular in outline, but roughly 30% of the bacteriocytes in the adult transiently bore protrusions or were elongated (figure 2*a*), and often changed shape, associated with net movement across the substratum (electronic supplementary material, video S1). Time-lapse observations of bacteriocytes revealed large changes in circularity (maximum value minus minimum value), averaging 0.18 ± 0.029 (mean ± s.e.m., 10 replicates), and migration distances of 18.5 ± 2.11 µm over 10 min test intervals (figure 2*b*–*d*). The regression of migration distance on circularity change was significant (figure 2*d*), indicating that shape changes and mobility of the bacteriocytes are linked.

To investigate the subcellular basis of the difference in mobility between nymph and adult bacteriocytes, we conducted a microscopical analysis of the bacteriocyte cytoskeleton. The bacteriocytes had a dense network of microtubules in cytoplasmic regions near to cell surfaces in contact with the external medium and peripheral actin filaments that were particularly pronounced at the interface between cells, especially in the nymphal bacteriocytes (figure 2*e*,*f*). To quantify this pattern, we compared pixel intensity in two peripheral locations, one facing external medium and one in contact with another bacteriocyte, for each of five replicate bacteriocytes. For microtubules, the pixel intensity was significantly greater in peripheral locations facing the external medium than those facing another bacteriocyte for both nymph bacteriocytes (*p* < 0.001) and adult bacteriocytes (*p* < 0.001); and a significant difference was also obtained for actin in nymph bacteriocytes (*p* < 0.005) but not for adult bacteriocytes (*p* > 0.05) (five replicates per test). The peripheral actin signal was also evident in bacteriocyte protrusions (figure 2*g*), suggesting that actin network remodelling in adult bacteriocytes is probably associated with shape changes and mobility of bacteriocytes.

**Figure 3.**
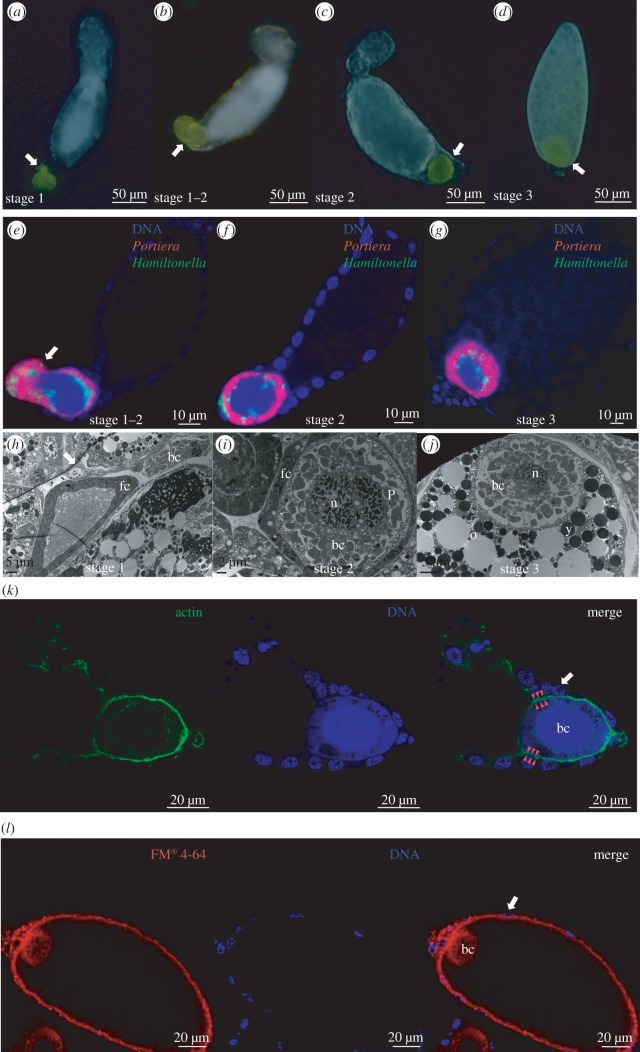
Transovarial transmission of bacteriocytes. (*a–d*) Three stages of bacteriocyte transmission identified by bright-field light microscopy; carotenoid pigments confer the natural green–yellow colour of bacteriocytes (white arrow denotes the bacteriocyte; note protrusion of bacteriocyte at stage 1). (*e–g*) Bacteriocyte transmission identified by FISH of *Portiera* (red) and *Hamiltonella* (green). DNA was stained by DAPI. Unmerged images are provided in electronic supplementary material, figure S2. The white arrow denotes the bacteriocyte with elongated shapes (*e*). (*h–j*) Transmission electron micrograph images: bc, bacteriocyte; fc, follicle cells bounding the developing oocytes; n, lobed nucleus of bacteriocyte; o, oocyte; P, *Portiera* cell; y, reserve substances in the oocyte cytoplasm. Note that each *Portiera* cell is bounded by an insect membrane (symbiosomal membrane), but does not fill the symbiosomal space in the EM because of differential shrinkage of bacterial and host cell contents during fixation. The white arrow denotes a wrinkle in the section (*h*). (*k,l*) Fluorescence microscopy of cytoskeleton and membranes with Hoechst counterstain (blue) for DNA. (*k*) Actin network (phalloidin-Alexa Fluor 488, green) bounding the bacteriocyte (bc, with red arrowheads) and basolateral regions of the follicle cells (white arrows) at stage 3. (*l*) Membrane structure (FM 4-64, red) of bacteriocytes (bc) and follicle cells (white arrows) at stage 3.

Our final analysis of the cellular dynamics of the bacteriocytes focused on the bacteriocytes transmitted to the ovaries of the adult female. Bacteriocytes were scored in three locations: external to the follicular sheath bounding each ovariole (figure 3*a*, stage 1), between the follicular sheath and the basal oocyte (figure 3*c*, stage 2) and bounded by the oocyte contents except at the posterior end (figure 3*d*, stage 3). Candidate instances of transition from stage 1 to stage 2 were observed regularly (figure 3*b*). The results of parallel FISH analysis and ultrastructural data are fully compatible with this interpretation, including confirmation of the presence of the symbiotic bacteria in the transmitted bacteriocytes (figure 3*e*–*j*; electronic supplementary material, figure S2). Complementary fluorescence microscopy revealed that bacteriocytes associated with ovaries were bounded by a peripheral cytoskeletal network of actin (figure 3*k*) and an intact cell membrane at stage 3 (figure 3*l*), providing definitive evidence that the intact maternal bacteriocyte is transmitted to the ovary. The fluorescent dye FM 4-64 used to visualize the cell membrane is also a convenient tracker of endocytosis [12] and the inclusion of the dye in cytoplasmic regions of the bacteriocyte (figure 3*l*) was indicative of sustained endocytic activity of the bacteriocyte cell membrane during vertical transmission.

In summary, our analysis revealed three major differences in the cell behaviour of bacteriocytes between nymphs and adult female insects: the bacteriocytes in nymphs were aggregated, while many adult bacteriocytes were isolated, mobile cells; cell proliferation in nymphs was associated with declining size but with a stable mean cell size over time in adults; finally, most of the bacteriocytes in the adult female insect were destined for transmission to the next generation, with up to 10% of the bacteriocytes in the adult insect associated with the ovaries at any time point.

### (b) Molecular basis of bacteriocyte dynamics and vertical transmission

We then investigated the molecular traits of the *B. tabaci* bacteriocytes by transcriptome analysis. Specifically, we compared the transcriptional profiles of host genes in bacteriocytes of fourth-instar nymphs and 7-day-old adult female insects, enabling us to identify candidate transcriptional patterns correlated with bacteriocyte traits associated with vertical transmission to the ovaries. The RNA-seq libraries yielded 54.5 and 52.4 million reads from nymph and adult bacteriocytes, respectively (electronic supplementary material, table S1). In total, 2,527 genes were differentially expressed: 1571 (62%) genes were upregulated and 956 genes downregulated in the adult bacteriocytes (electronic supplementary material, dataset S1a), including 1187 (47%) with KEGG Orthology (KO) definition and 666 genes with Gene Ontology (GO) term(s).

**Figure 4.**
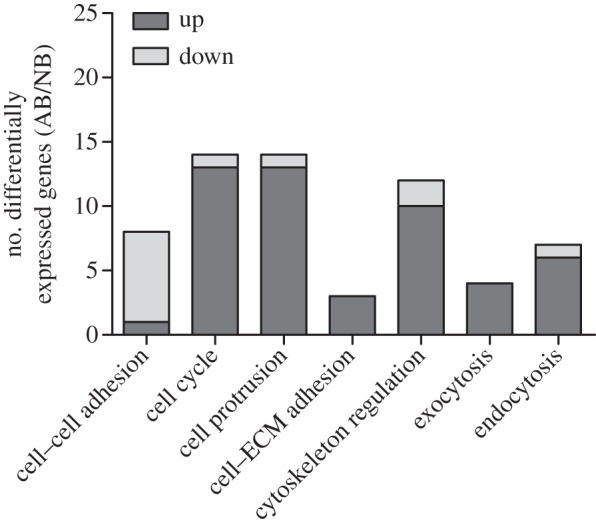
Gene expression involved in cellular processes in adult bacteriocytes (AB) compared with nymph bacteriocytes (NB). Genes contributing to each functional class are listed in electronic supplementary material, database S1b.

Our global analysis of the transcriptome data focused on genes with biological process classified as ‘cellular process’ (electronic supplementary material, figure S3). Pathway and GO analysis of this gene set revealed 62 genes differentially expressed between the adult and nymph bacteriocytes (figure 4; electronic supplementary material, datasets S1a and S1b). Genes with enriched expression in the nymph bacteriocytes have predicted functions in cell–cell adhesion, congruent with the tight association between bacteriocytes in nymphs (but not adults; figures 1*b* and 2*e*). Genes with elevated transcript abundance in adult bacteriocytes relate to cell division, cell cycle regulation, cell motility, and cell adhesion to extracellular matrix (ECM) and endocytosis/exocytosis, consistent with changes in the pattern of cell cycle and onset of cell mobility in the adult bacteriocyte (figures 1*c* and 2*a*–*d*,*g*). The expression of genes with predicted endocytic/exocytic activity is indicative of vesicle-mediated translocation of nutrients or signals between bacteriocytes and external medium, consistent with the high abundance of microtubules, which mediate intracellular vesicular transport, in the bacteriocyte near the external surface (figure 2*e*,*f*), and the direct evidence for endocytosis of the membrane dye FM 4-64 (figure 3*l*). The elevated endocytosis/exocytosis in adult bacteriocytes may be linked to the smaller size and larger surface area in relation to volume available in the external medium.

## 4. Discussion

This study demonstrates major differences in cellular behaviour and gene expression patterns between bacteriocytes in adult females and nymphs of the whitefly *B. tabaci* that can reasonably be inferred to underpin the observed pattern of symbiont transmission in the adult (see Introduction). Two key traits of bacteriocytes in the adult host are: the sustained proliferation of bacteriocytes, driven by the demand that each oocyte is provided with a single bacteriocyte and the onset of bacteriocyte mobility in the adult, for transfer to the ovaries.

The proliferating bacteriocyte population through much of the lifespan of the insect host is underlain by dramatic differences in cellular organization between the bacteriocytes in the nymph and adult insect. Most distinctively, the bacteriocytes of nymphs are adherent with divisions at progressively smaller sizes, while the adult bacteriocytes occur as loose aggregates or isolated cells with uniform size distributions. These data are indicative of stage-dependent differences in the regulation of cell division, perhaps with greater significance of a timer-control in nymphs and sizer-control in adults [13]. The cell–cell adherence of nymph bacteriocytes can be linked to the elevated expression of multiple cell adhesion genes in these cells and may function to mediate the efficient transfer of nutrients between cells. Interestingly, the bacteriocyte aggregations comprise no more than 10–12 cells, and part of the surface of every cell is exposed to the haemolymph, enabling direct exchange of nutrients with the haemolymph. However, the progressive increase in total volume of the bacteriocytes through much of the adult lifespan (when the insects are not increasing in size) suggests that the bacteriocytes also play an important role in supporting adult insect function, most probably through the bacterial synthesis of essential amino acids that contribute to insect protein synthesis [9,10].

Detailed descriptions of the patterns of bacteriocyte size and number are available for one other insect bacteriocyte symbiosis: the association between aphids and the bacterium *Buchnera aphidicola*. The increase in bacteriocyte biomass by balanced cell growth and division in the whitefly is in striking contrast with a related insect group, the aphids, in which bacteriocytes divide 2–3 times in the embryo and subsequently increase exclusively by cell growth associated with extensive genomic endoreduplication [14,15]. The different developmental patterns of bacteriocyte growth and division in different insect groups can be attributed to the difference in pattern of symbiont transmission. Although intact bacteriocytes are transmitted in the whitefly system, isolated bacterial cells transit from maternal bacteriocytes to the ovaries of aphids [16]. The different bacteriocyte dynamics between whiteflies and aphids have potentially large implications for symbiosis function. While the high surface area–volume relationship of the small bacteriocytes of whiteflies facilitates nutrient transfer between the bacteriocyte and other insect organs, the non-dividing aphid bacteriocyte does not incur the likely cost of reduced metabolic activity during mitosis and cell division [17].

The second key difference between bacteriocytes in adult and nymphs of *B. tabaci* was the mobility of adult cells, associated with the disaggregation of the bacteriocytes in the pupal/early adult insect. This profound change in cell behaviour co-occurs with increased expression of genes with cytoskeletal function and cell–ECM interactions. These changes are fully congruent with the transfer of bacteriocytes to the ovaries for vertical transmission. Our study confirms and extends published evidence that the bacteriocytes crawl between cells of the follicular epithelium [3,5] to reside external to the oocyte membrane. The strict allocation of a single bacteriocyte to the posterior pole of every oocyte in *B. tabaci* suggests that transmission is tightly orchestrated, and may involve specific chemical communication of the bacteriocytes with the follicle cells and oocyte. Communication may be initiated from a distance, perhaps via directed bacteriocyte movement towards a diffusible chemical released from oocytes, and indications that the bacteriocytes may continue as active participants in the exchange of nutrients and signalling molecules come from the very high rates of energetically demanding endocytic activity (figure 3*l*) by bacteriocytes after transovarial transmission is complete.

In summary, this study has revealed that symbiosis transmission in the whitefly *B. tabaci* is associated with fundamental cellular remodelling of the bacteriocyte, involving coordinated changes in patterns of cell division, cell adhesion and mobility, together with major changes in gene expression. We hypothesize that, although the morphology of the transmission process varies among different animal groups, there will be some commonalities in underlying mechanism, in terms of controls over cellular behaviour and gene expression. Other aspects of transmission may be unique to each host bacterial system.

The focus of this study, *B. tabaci*, is a globally important insect pest, linked to its capacity to vector devastating plant viruses in multiple crops and increasing incidence of insecticide resistance. This major pest is absolutely dependent on the symbiotic bacteria in the bacteriocytes. The elucidation of the cellular and molecular processes underlying transovarial transmission provides the foundation for identifying specific molecular targets for disruption of transmission, as a novel control strategy for *B. tabaci* and other major whitefly pests.

## Supplementary Material

Figure S1-S3

## Supplementary Material

Table S1

## Supplementary Material

Dataset S1

## Supplementary Material

Dataset_S2

## Data accessibility

The raw reads are available at the National Center for Biotechnology Information (NCBI) Short Read Archive (SRA) with accession numbers SRR1523521 and SRR2001505 for adult and nymph bacteriocytes, and the assembled sequences are in the NCBI Transcriptome Shotgun Assembly database (TSA) database under the accession numbers GBII00000000 and GDAA00000000 for adult and nymph bacteriocytes. The datasets supporting this article have been uploaded as part of the electronic supplementary material. The adult RNA-seq data have been used in a different context by Luan *et al.* [9].
