# Supplementary figures and images for: Cellular and molecular remodelling of a host cell for vertical transmission of bacterial symbionts
Source: Proc Biol Sci. 2016 Jun 29;283(1833):20160580. doi: 10.1098/rspb.2016.0580 (PMC4936034; doi:10.1098/rspb.2016.0580)

**Figure S1**

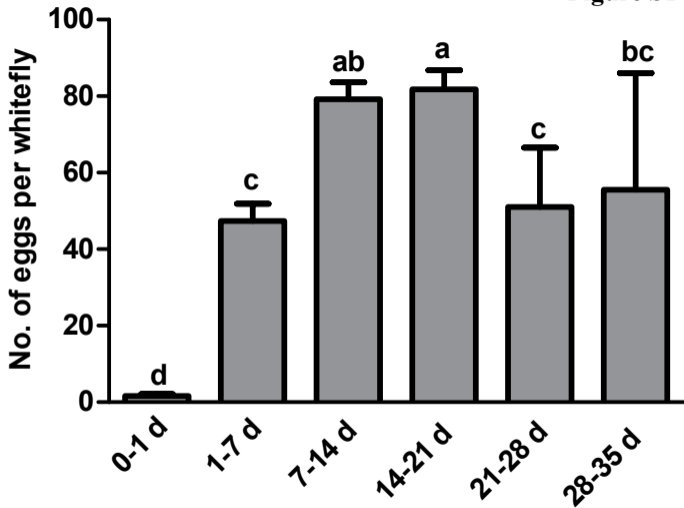

Figure S2

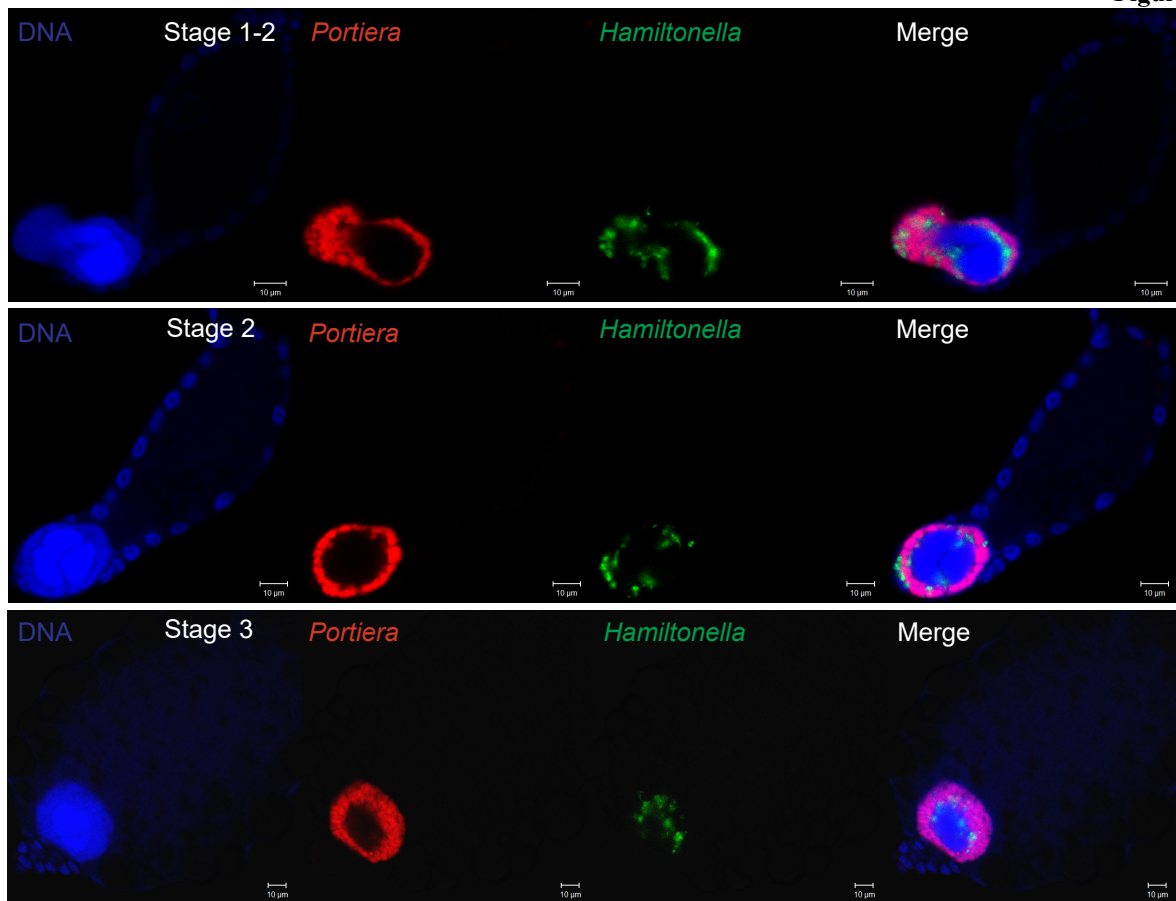

Figure S3

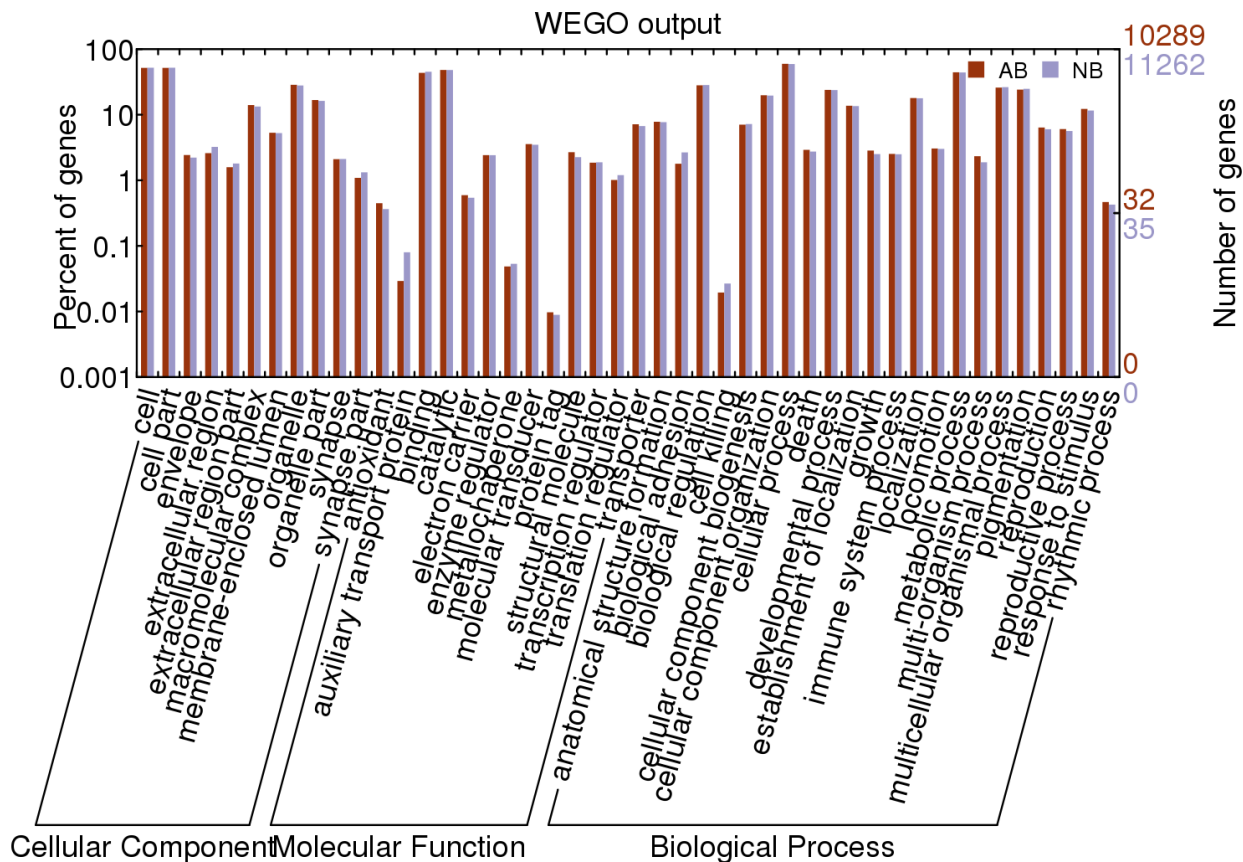

Supplement: Figure S1-S3 [file rspb20160580supp1.pdf]
