# Supplementary material for: Cellular and molecular remodelling of a host cell for vertical transmission of bacterial symbionts
Source: Proc Biol Sci. 2016 Jun 29;283(1833):20160580. doi: 10.1098/rspb.2016.0580 (PMC4936034; doi:10.1098/rspb.2016.0580)
Supplement: Table S1 [file rspb20160580supp2.docx]

**Table S1 Summary statistics of RNA-Seq of nymph bacteriocytes and adult bacteriocytes**

| *Category* | *Value for mRNA-Seq library* | | |
| --- | --- | --- | --- |
|  | *Nymph bacteriocyte* | *Adult bacteriocyte*^1^ |  |
| Total Raw Reads | 57,579,762 | 55,421,140 |  |
| Total Clean Reads | 54,466,828 | 52,385,202 |  |
| Total Contig Number | 111,437 | 97,534 |  |
| Total Contig Length(nt) | 53,292,921 | 51,299,889 |  |
| Total Unigene Number | 57,143 | 51,479 |  |
| Total Unigene Length(nt) | 69,769,973 | 67,638,283 |  |
| Distinct Singletons | 40,960 | 36,143 |  |

^1^ This transcriptome has been published previously in Luan, J.-B., Chen, W., Hasegawa, D.K., Simmons, A.M., Wintermantel, W.M., Ling, K.-S., Fei, Z., Liu, S.-S. & Douglas, A.E. 2015 Metabolic coevolution in the bacterial symbiosis of whiteflies and related plant sap-feeding insects. *Genome Biol Evol* **7**, 2635–2647. (doi:10.1093/gbe/evv170).
